# Case Report: A Case of Myeloproliferative Neoplasm Complicated by Alopecia Areata

**DOI:** 10.3389/fmed.2022.895699

**Published:** 2022-05-26

**Authors:** Yotaro Tamai, Shinichi Teshima, Shun Tsunoda, Wataru Kamata, Shuku Sato

**Affiliations:** ^1^Division of Hematology, Shonan Kamakura General Hospital, Kamakura, Japan; ^2^Division of Pathology, Shonan Kamakura General Hospital, Kamakura, Japan

**Keywords:** myeloproliferative neoplasm, alopecia areata, essential thrombocytosis, JAK, ruxolitinib

## Abstract

Myeloproliferative neoplasms (MPNs) are caused by genetic abnormalities in the stem cells and manifest with various systemic symptoms. Here, we describe a case of MPN complicated by alopecia areata. A 51-year-old woman visited our hematology department for further evaluation of a slight platelet elevation. Her recent medical history included 3 years of concurrent severe alopecia, mild fatigue, and hot flashes but no fever and weight loss. Physical examination revealed unilateral hair loss on the entire body but no hepatosplenomegaly. Laboratory analysis revealed a normal hemoglobin level, normal white blood cell count, and platelet count of 377,000/μL. Genetic testing confirmed the presence of the *JAK2* V617F mutation. Bone marrow examination revealed no morphologic dysplasia in any stem cell lineage and no fibrotic change. Skin biopsy revealed lymphocyte infiltration around the hair follicles. We diagnosed MPN, unclassifiable, which was believed to be the cause of alopecia. About 6 months after treatment with ruxolitinib began, the patient's hair growth dramatically improved. The differential diagnosis of MPNs should include hematological diseases when affected patients have alopecia areata.

## Introduction

Myeloproliferative neoplasms (MPNs), which include essential thrombocythemia, polycythemia vera, and myelofibrosis, are characterized by various systemic manifestations. Results of an online survey of 1,179 patients with MPNs indicated that constitutional symptoms and symptoms associated with splenomegaly were prominent and compromised the patients' quality of life (1). Additional complaints included fatigue, night sweats, bone pain, fever, and weight loss. These systemic symptoms are characteristic of MPNs, but associated hair loss has not been previously reported. Alopecia areata (AA) is a T cell-mediated autoimmune disease characterized phenotypically by hair loss and histologically by hair follicle bulbs surrounded by infiltrating T cells (2). The inflammatory assault on anagen follicles induces a premature conversion to catagen, which results in a persistent telogen phase in which the hair shaft has already been shed, as manifested by the telogen germinal unit. Ruxolitinib is an inhibitor of activated JAK1 and JAK2 that is approved for the treatment of myelofibrosis (3). Dysregulated activation of Janus kinase/signal transducer and activator of transcription (JAK-STAT) signaling is implicated in the uncontrolled proliferation of hematopoietic progenitors and the generation of a proinflammatory reaction responsible for systemic symptoms (4). It has been shown that the anti-inflammatory action of ruxolitinib is effective against other autoimmune diseases, including AA (5).

Here, we describe a case of MPNs in which AA developed. Ruxolitinib, which was started for MPN treatment, was successful in alleviating AA, and AA was thought to have developed in association with the MPNs. This is the first report of AA as a manifestation of MPNs.

## Case Presentation

**Figure 1:**
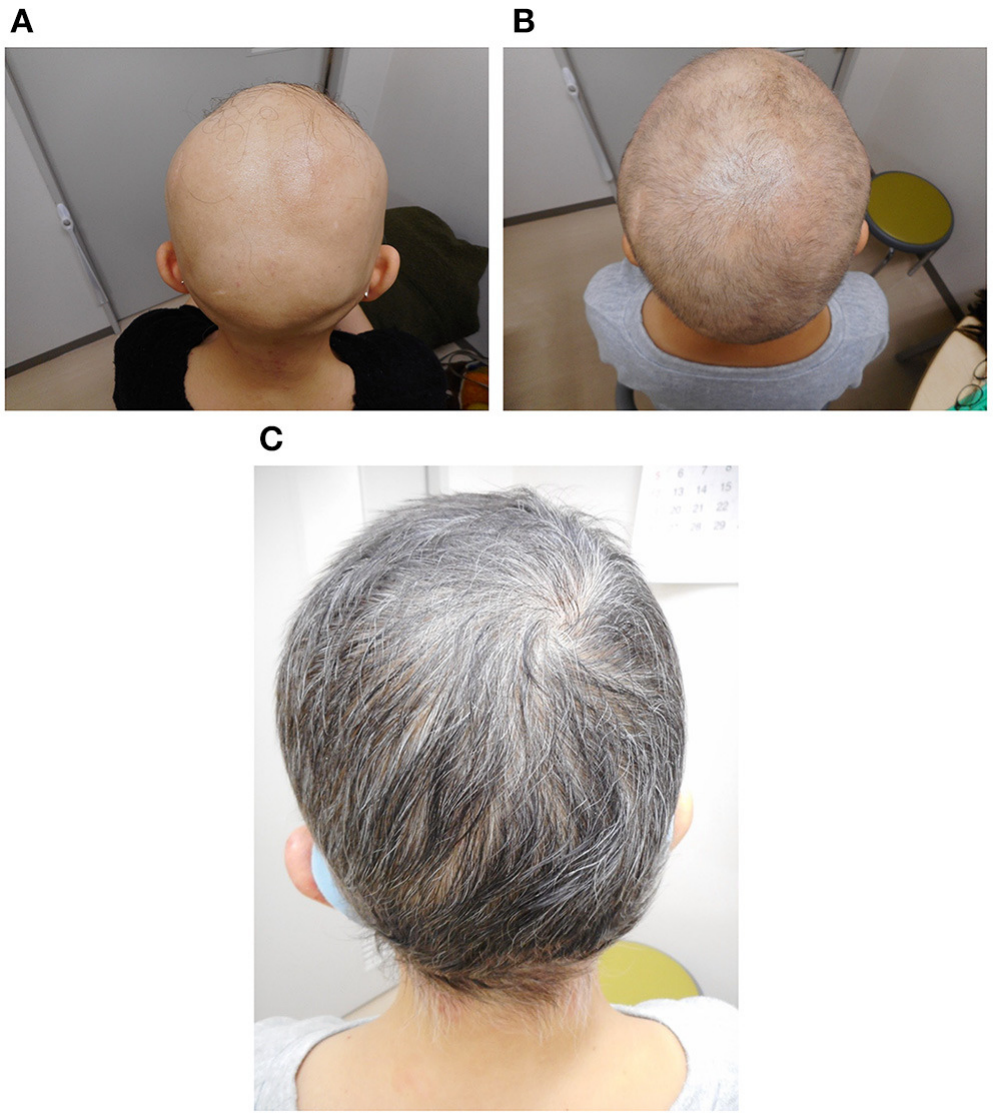
Photographs of the patient's scalp before treatment **(A)**, 6 months after the start of treatment **(B)**, and 1 year after the start of treatment **(C)**.

**Figure 2:**
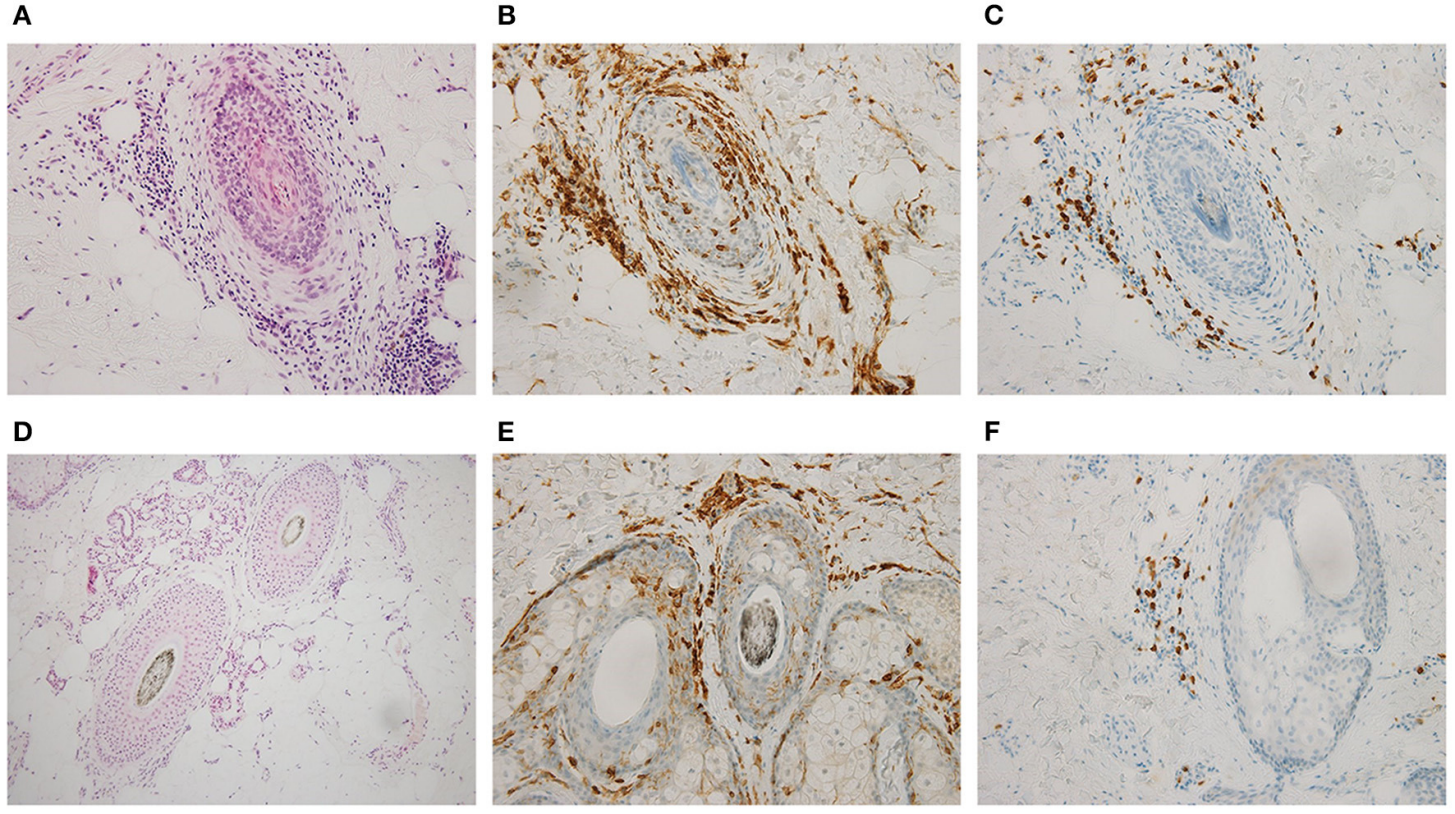
Skin biopsy of the scalp findings before **(A–C)** and 1 year after ruxolitinib treatment began **(D–F)**. Hematoxylin and eosin stains (**A,D**; ×200) and immunohistochemical stains for CD4 (**B,E**; ×200) and CD8 (**C,F**; ×200) showed that infiltrative CD8-positive T cells disappeared after treatment.

A 51-year-old woman visited our hematology department for further evaluation of a slight platelet elevation. The patient was healthy and was not prescribed any medication; however, the patient's recent medical history included 3 years of severe AA with mild fatigue and hot flashes but no fever or weight loss. On physical examination, the patient exhibited hair loss on her head and one entire side of her body (Figure 1A) but no hepatosplenomegaly. The patient's laboratory results were as follows: hemoglobin level, 13.6 g/dL (reference range: 13.1–17.6 g/dL); white blood cell count, 6,300/μL (reference range: 3,000–9,700/μL); and platelet count, 377,000/μL (reference range: 124,000–305,000/μL). Thyroid function and iron and ferritin levels were normal, and tests for rheumatoid arthritis factors and antinuclear antibody yielded negative results. She had no risk of thrombotic events, such as hypertension, smoking history, diabetes, or dyslipidemia. Genetic testing with peripheral blood confirmed the presence of the *JAK2* V617F mutation and the absence of BCR/ABL fusion protein and CALR and MPL mutations. The results of bone marrow biopsy revealed a slightly increased megakaryocyte count, a normoplastic marrow with a myeloid/erythroid ratio of 2:3, and no morphologic dysplasia of megakaryocyte, suggesting prefibrotic/early primary myelofibrosis. G-band analysis revealed no chromosomal abnormalities or translocations. Based on these findings, myelodysplastic syndrome was excluded in differential diagnosis. Skin biopsy revealed peribulbar lymphocytic inflammation (the “swarm of bees” sign; Figure 2A). Immunostaining showed both CD4- and CD8-positive lymphocytes (Figures 2B,C).

The patient was diagnosed with myeloproliferative neoplasm, unclassifiable (MPN-U), on the basis of the 2017 World Health Organization classification; we also thought that this was the cause of AA. Although we found no evidence of myelofibrosis, the hot flashes and fatigue were considered systemic symptoms associated with MPNs, and treatment with the JAK inhibitor ruxolitinib (starting at 10 mg daily and gradually increasing to 30 mg daily) was initiated. The hot flashes and fatigue were quickly alleviated. About 6 months after the start of ruxolitinib treatment, signs of improvement in other symptoms were observed, as was hair growth (Figure 1B); after 1 year, the improvement was dramatic (Figure 1C). Skin biopsy of the scalp at that time confirmed hair in multiple hair follicles, and the numbers of infiltrating lymphocytes around the hair roots were reduced (Figure 2D). CD4-positive lymphocytes were few and scattered, but almost no CD8-positive lymphocytes were observed (Figures 2E,F).

With continued ruxolitinib treatment, hair loss was no longer observed, and other body hair, including the eyebrows, regrew. The platelet count steadily increased to 580,000/μL over 1 year. The daily dose of ruxolitinib remained at 40 mg/day, and the diagnosis was changed from MPN-U to essential thrombocythemia (ET). The laboratory results at the time of ET diagnosis were as follows: hemoglobin, 12.2 g/dL; white blood cell count, 5,600/μL; and platelet count, 451,000/μL. The revised International Prognostic Score for Thrombosis in ET (IPSET-thrombosis) score was low (6).

## Discussion

MPN can manifest with numerous clinical symptoms, which require careful evaluation (7). In the current patient, the platelet count was initially low, suggesting MPN, but increased over time to meet the diagnostic criteria of ET (>450,000/μL). The platelet count threshold for ET diagnosis (>600,000/μL) (8) was later revised based on case reports of thrombosis even in patients with low platelet counts. The presence of *JAK2* V617F mutation excludes reactive thrombocytosis (9), and the diagnostic subgroup of MPN-U, defined in the 2017 WHO classification, includes patients who do not sufficiently meet the diagnostic criteria, i.e., do not present the characteristic features. To the best of our knowledge, only one case of successful ruxolitinib treatment for ET complicated by AA has been reported (10), but AA as a symptom of MPNs has not been previously reported. That report and ours referred to separate diseases, but we considered the hair loss to have the same pathogenesis.

The previous report suggested that CD8- and NKG2D-positive T cells are both necessary and sufficient for AA in a mouse model (5). One year after our patient started treatment, CD4-positive lymphocytes were still observed, but barely any CD8-positive lymphocytes were present, which is consistent with the findings in the previous report. AA is a well-known complication of autoimmune diseases, such as thyroid disease, rheumatoid arthritis, and systemic lupus erythematosus (11), but these diseases were not observed in our patient; only MPNs were present. Recently, in patients with AA, the JAK-STAT signaling pathway has been shown to be a possible therapeutic target, and thus, interest in treatment with JAK inhibitors has increased (5). No report of AA so far has mentioned the possibility of MPNs and testing for *JAK* mutations.

JAK2 V617F and other mutations in MPNs are responsible for upregulated JAK-STAT signaling (12). Panteli et al. demonstrated that patients with ET had increased serum levels of interleukin (IL)-2 and soluble IL-2 receptor α, which may suggest chronic inflammation caused by increased lymphocyte activity (13). Brajac et al. had previously demonstrated that IL-2 receptor α was expressed on infiltrating lymphocytes surrounding human AA hair follicles (14). Moreover, the JAK-STAT pathway affects regulatory T cells (Tregs) and IL-17 expression. Yang et al. demonstrated that IL-6 upregulates the expression of IL-23, which together with IL-6 activates STAT3 and hence promotes the development of T helper 17 cells (15). This differentiation was severely impaired in STAT3-deficient cells, which exhibited reduced expression of retinoid acid receptor-related orphan receptor γ (RORγt) and IL-17 and increased levels of Tregs. André et al. noted that major intracellular signaling pathways, including the JAK-STAT pathway, play a role in the chronic activation of antigen-presenting cells (16); these cells can escape suppression by Tregs and generate activated T cells that are refractory to suppression by Tregs (17). Although these findings have not been examined in detail, it is possible that constitutive activation of the JAK-STAT pathway in MPNs contribute to the development of AA.

It is important to consider the indications for thrombosis prophylaxis using aspirin or cytoreductive therapy in patients with ET. The current patient harbored a *JAK2* mutation. However, she was at low risk of thrombosis based on the revised IPSET-thrombosis and her platelet count was not high; therefore, she was not administered aspirin. Ruxolitinib has shown a certain extent of efficacy in primary myelofibrosis, such as symptomatic improvement, reduction of splenomegaly, and improved prognosis (18). On the other hand, albeit not confirmed, the effectiveness of ruxolitinib in ET was suggested to attenuate symptomatic improvement (19). Multiple clinical trials of AA treatment with JAK inhibitors, including ruxolitinib, have started to show the potential of these drugs (20). Also in our patient, ruxolitinib, which was started as treatment for MPNs, may have also ameliorated AA, but both conditions are characterized by lymphocyte abnormality mediated by the JAK-STAT pathway; furthermore, we interpreted hair loss as a manifestation associated with MPNs. This hypothesis is based on insights from multiple basic research studies, but the fact that AA occurred with MPNs indicates that some patients with AA may also have MPNs.

In summary, our patient with MPNs also developed AA, and the hair loss was reversed by MPN treatment. AA resulting from T cell infiltration of the hair follicle may be a pathognomonic manifestation of MPNs. Thus, the possibility of hematological disease should be considered in patients with AA and any minor abnormalities in complete blood count; the confirmation of this possible association requires future studies.

## Data Availability Statement

The original contributions presented in the study are included in the article/supplementary material, further inquiries can be directed to the corresponding author/s.

## Ethics Statement

Ethical review and approval was not required for the study on human participants in accordance with the local legislation and institutional requirements. The patients/participants provided their written informed consent to participate in this study. Written informed consent for the publication of potentially identifiable data/images was obtained from the patient(s).

## Author Contributions

YT wrote the original draft and treated the patient. YT and STe created the figures. YT, STs, WK, and SS contributed to diagnosis. All the authors contributed to the manuscript revision, read, and approved the manuscript.

## Conflict of Interest

The authors declare that the research was conducted in the absence of any commercial or financial relationships that could be construed as a potential conflict of interest.

## Publisher's Note

All claims expressed in this article are solely those of the authors and do not necessarily represent those of their affiliated organizations, or those of the publisher, the editors and the reviewers. Any product that may be evaluated in this article, or claim that may be made by its manufacturer, is not guaranteed or endorsed by the publisher.
